# The challenges of *Plasmodium vivax* human malaria infection models for vaccine development

**DOI:** 10.3389/fimmu.2022.1006954

**Published:** 2023-01-05

**Authors:** Wanlapa Roobsoong, Anjali Yadava, Simon J. Draper, Angela M. Minassian, Jetsumon Sattabongkot

**Affiliations:** ^1^ Mahidol Vivax Research Unit, Faculty of Tropical Medicine, Mahidol University, Bangkok, Thailand; ^2^ Biologics Research & Development, Walter Reed Army Institute of Research, Silver Spring, MD, United States; ^3^ Department of Biochemistry, University of Oxford, Oxford, United Kingdom

**Keywords:** *Plasmodium vivax in vitro* culture, controlled human malaria infection, CHMI, malaria human challenge study, *P. vivax* vaccine, Malaria Infection Study Thailand, MIST

## Abstract

Controlled Human Malaria Infection models (CHMI) have been critical to advancing new vaccines for malaria. Stringent and safe preparation of a challenge agent is key to the success of any CHMI. Difficulty producing the *Plasmodium vivax* parasite *in vitro* has limited production of qualified parasites for CHMI as well as the functional assays required to screen and down-select candidate vaccines for this globally distributed parasite. This and other challenges to *P. vivax* CHMI (*Pv*CHMI), including scientific, logistical, and ethical obstacles, are common to *P. vivax* research conducted in both non-endemic and endemic countries, with additional hurdles unique to each. The challenges of using CHMI for *P. vivax* vaccine development and evaluation, lessons learned from previous and ongoing clinical trials, and the way forward to effectively perform *Pv*CHMI to support vaccine development, are discussed.

## Introduction

Inducing human challenge by inoculation with malaria-infected blood was first used as a treatment (malariotherapy) for neurosyphilis in Europe and the United States in the early 1900s (1, 2).

More recently, Controlled Human Malaria Infection (CHMI) has been applied to the fields of malaria vaccine and drug development. The advent of *in vitro* culture methods for *P. falciparum* in the mid-1970s exponentially expedited studies on several aspects of *in vitro* research, one of which is the use of these cultured parasites in human infection studies (3, 4). By 1986, investigators at WRAIR published the first report on *Pf*CHMI using mosquitoes infected with gametocytes from *in vitro* cultured parasites (5). Worldwide, thousands of healthy trial participants have been infected with *P. falciparum* sporozoites (6, 7) and more than 500 with blood-stage parasites (8–13). *Pf*CHMI is now well established in both non-endemic countries and numerous African trial sites and is an important tool in the rapid assessment and down-selection of candidate antimalarial drugs and vaccines. The re-establishment of mosquito-bite induced *Pv*CHMI under current ethical and regulatory guidelines was initiated in the mid-2000s. As opposed to *Pf*CHMI, where using laboratory-cultured gametocytes is feasible, the source of *P. vivax* gametocytes for infecting mosquitoes is naturally-infected humans. Until 2018 there had only been a handful of published studies in three areas of the world, Colombia, USA and Australia (14–22), and no experience in Europe until a group in Oxford (UK) was the second globally to produce a cryopreserved bank of a *P. vivax*-infected blood suitable for *Pv*CHMI (23). McCarthy and team were the first to use this technique to evaluate new drugs for *P. vivax* in healthy volunteers in Australia (14). This article summarizes the challenges and progress with continuous culture of *P. vivax*, the different challenge protocols (mosquito bite & blood stage infection), and the ethical and logistical issues in setting up *Pv*CHMI models for *P. vivax* vaccine development.

## Continuous culture of *P. vivax* – An update

*P. vivax* has raised the bar when it comes to difficulties in conducting robust CHMI studies, particularly due to the lack of a continuous parasite culture method. Finding culture conditions that could support asexual propagation while maintaining productive gametocyte production would impact hugely on the time required to develop effective vaccines and drugs. The lack of a continuous *in vitro* culture system has thus long-hampered an in-depth understanding of this parasite’s biology. Together, these obstacles have challenged the development of functional assays with which to screen and down-select candidate vaccines and drugs. This includes *in vitro* assays of growth inhibition activity (GIA) using cultured blood-stage parasites, widely used in the *P. falciparum* field to screen for functional antibody responses. Consequently, this has delayed the identification of optimal combinations of blood-stage antigens that could be targeted to successfully inhibit *P. vivax* blood-stage growth by vaccination. Several groups have recently succeeded in establishing short-term *P. vivax* culture for invasion inhibition assays using enriched reticulocytes from cord blood (24), but such methods are still dependent on access to fresh *P. vivax* isolates from patients, limiting the routine use of such assays to endemic regions. Moreover, in the absence of a *P. vivax* blood-stage culture system that can also yield gametocytes, the production of infected mosquitoes for sporozoite- and/or transmission-stage studies (25) also requires access to blood samples from *P. vivax* patients (14, 23). Filling this gap will be key to spear-heading *P. vivax* research and vaccine development.

Since the success of *P. vivax in vitro* culture using the Chesson strain adapted from non-human primate to human blood (26), several attempts to grow the *P. vivax* parasite exponentially *in vitro* have relied on two key factors: the culture micro-environments (26–30) and host reticulocytes (26–34). Reticulocytes derived from hematopoietic stem cells and immortalized erythroid progenitors have been shown to support *P. vivax* maturation (29, 32, 34–36); however, the production cost is still high and only small-scale production has been achieved. The culture microenvironments have direct impacts not only on parasite development but also stabilize the healthiness of the reticulocyte. Among different culture conditions and different sources of reticulocyte that have been tried (26, 28–30, 32–34, 37), none of these two-dimensional systems could lead to exponential growth nor reliable infective gametocyte production. The transcending progression from two-dimensional (2D) to three-dimensional (3D) culture systems, which mimic the microenvironment of the desired functional organ (38–40), could fuel the progression of the continuous culture of *P. vivax* blood stage. The 3D human bone marrow, which exhibits the structural features of human bone marrow while supporting the maintenance of hematopoietic stem cells (39), can be further utilized for *P. vivax* culture. On the other hand, progress has been made on vivax research using humanized mouse models. Two humanized mice models have been used to propagate *P. vivax* erythrocytic stage successfully (41–43). In the human liver-chimeric mouse model (huHep mouse), the mouse liver has been repopulated with human hepatocytes, and has been shown to support the complete exo-erythrocytic stage development of *Plasmodium spp (* 42, 44). This huHep mouse model has been further utilized for *P. vivax* by infusing the human reticulocytes, allowing the exo-erythrocytic merozoites to invade and develop to erythrocytic stage, including gametocytes (43). The recently developed Human Immune System Human Erythrocyte mouse model (HIS-HEry), repopulated with human erythropoietic progenitors in mouse bone marrow, provides robust circulating human reticulocytes which support the *in vivo* propagation of *P. vivax* erythrocytic stage and importantly infective gametocytes (41). The passive transfer of *P. vivax* infected blood from the donor HIS-HEry infected mouse to the recipient uninfected mouse allows the continuous *in vivo* propagation of this parasite. These advances in 3D culture systems and humanized mouse models have enlightened *P. vivax* research, and drug and vaccine development.

## How to challenge volunteers using CHMI: Mosquito-bite or blood-stage challenge model?

**Figure 1:**
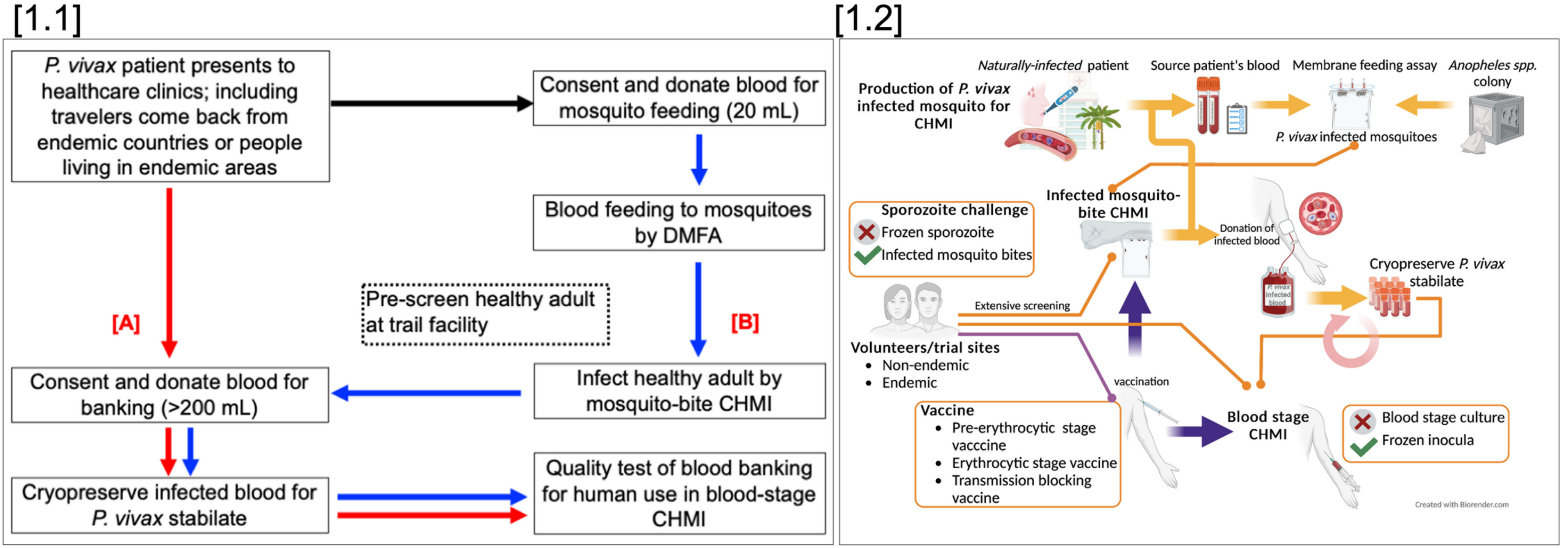
Overview of *Pv*CHMI to support vaccine development; [1.1] Strategies to produce a *P. vivax* infected blood bank. *P. vivax* infected blood can be obtained from *P. vivax* infected travelers returning from endemic areas or from patients seeking treatment at health facilities in endemic areas. Collected blood, after the informed consent process, is used to prepare blood banking directly (>200 mL) **(A)**, or fed to laboratory-reared mosquitoes through a direct membrane feeding assay (DMFA) to produce *P. vivax* infected-mosquitoes (less blood volume required for membrane DMFA). In parallel, healthy volunteers must be identified, pre-consented for mosquito-bite CHMI and a blood donation. Shipment of infected mosquitoes from the field site must then be critically timed to infect the donor volunteer(s) *via* mosquito-bite CHMI **(B)**. These volunteers are then monitored closely for blood donation to produce a *P. vivax* stabilate. [1.2] Utilization and challenges of different *Pv*CHMIs. Sporozoite-induced CHMI can be done through mosquito bite-challenge but not injection of sporozoites. Mosquito bite-CHMI can be used to evaluate all stages of vaccines including pre-erythrocytic stage vaccines, blood-stage vaccines, and transmission-blocking vaccines. Blood-stage induced CHMI can be used to evaluate blood-stage and transmission-blocking vaccines.

The type of *P. vivax* CHMI model (initiated by mosquito-bite delivered sporozoites or direct blood-stage inoculation) chosen for a particular clinical study will depend on the aspect of immunity that is being interrogated, and/or the intervention being tested (drug or vaccine) and/or the lifecycle stage against which these are active. An overview of challenge agent production for mosquito-bite and blood-stage *Pv*CHMI is summarized in **Figure 1**.

One advantage of mosquito-bite challenge is that it mimics the route of natural infection. However, it requires access to infected patients to initiate the production of infected mosquitoes and the constant production of mosquitoes, as well as relevant entomological expertise. There are also substantial logistics associated with the shipment of mosquitoes, safety testing of the donor blood sample(s) and timing with carefully pre-planned vaccination studies usually occurring in other locations or countries. It also inevitably leads to the use of genetically variable parasite isolates, and the issue of hepatic dormancy and potential relapse has to be accounted for when planning any clinical trial involving human participants (18).

In contrast, a blood-stage *P. vivax* CHMI model involves intravenous injection of blood-stage *P. vivax* parasites. Infected participants are monitored for the development of symptoms and blood-stage infection by daily qPCR in real-time. The parasite multiplication rate (PMR) is modeled from the qPCR data and the impact on PMR is usually the primary endpoint measure of vaccine “efficacy” for blood-stage vaccine candidates. A blood-stage CHMI model has been the preferred model to test blood-stage vaccines for *P. falciparum* (9, 45). This also offers a more feasible approach for *Pv*CHMI studies in a non-endemic setting, and aspects of these studies can be more easily standardized. In particular, this approach can allow the delivery of multiple studies with the same challenge strain of parasite, and the same inoculum size can be administered to each participant (9, 12). Because this route of infection bypasses the liver stage, there is no (known) risk of *P. vivax* relapse. However, *P. vivax* blood-stage inocula are not widely available.

## Establishment of infected mosquitoes for *Pv*CHMI

### Regulatory, logistical and ethical concerns

Regulatory requirements for conducting CHMI are stringent and vary according to the country in which they are being performed. Because there are no cultured lots of *P. vivax*, parasites need to be obtained from infected human donors. Therefore, there are additional regulatory, logistical and ethical considerations in conducting *Pv*CHMI. Among the more important considerations are the potential for inadvertent transmission of adventitious agents from the donor to the recipient and the potential of relapse due to inadequate treatment of dormant hypnozoites. To ameliorate these concerns, the donor and recipient inclusion and exclusion criteria for *Pv*CHMI differ from those of *Pf*CHMI and are discussed below.

### *Plasmodium vivax*-infected patient (donor) blood screening

After the bleeding of patients for mosquito feeding, a blood sample needs to be screened for blood- and vector-borne infections, due to the potential risks associated with the inadvertent transmission of such infections to subsequent healthy volunteers. All centers therefore screen the donors for blood borne diseases as per national blood and transplant guidelines. The main screening for blood-borne pathogens is similar in all trials. The different screening is usually related to mosquito-borne diseases that vary between regions/countries. For example, there is no local transmission of Chagas disease in Thailand, so that blood screening for this disease is not required while it transmits locally in Colombia; thus, the study will require screening for this disease in blood donors. Knowledge of infections with the potential for transmission by *Anopheles* spp. mosquitoes in these settings is an important consideration. Although *Anopheles* spp. mosquitoes are the primary vector for the transmission of malaria, some are known to transmit lymphatic filariasis and may serve as vectors for certain endemic viral infections (46). The mosquitoes for CHMI are derived from qualified laboratory-reared colonies, in order to minimize the transmission risk of other infections from the patient’s blood (on which the mosquito has fed), region-specific vector-borne testing has been undertaken by all centers, including, but not limited to, Filaria, Chikungunya, Japanese encephalitis, Dengue, Zika and West Nile viruses. The list of mosquito-borne diseases for each country is available from the Ministry of Public Health and local CDC. The safety of the volunteers from the other mosquito-borne diseases after challenge by mosquito bite is the major concern for local IRBs in all countries conducting the trial. The highly qualified mosquito is an important key for success of sporozoite-*Pv*CHMI and can be evaluated from blood feeding rate and mortality rate, besides they must be free of other mosquito borne diseases. The high-quality vector colony usually does not depend on the number of years the colony has been established but rather on the staff experiences.

## Healthy volunteer inclusion/exclusion criteria

Inclusion and exclusion criteria for volunteers in CHMI studies are usually rigorous, but for mosquito-bite delivered *P. vivax* malaria there are a few added complexities. The susceptibility of the mosquito-bite recipients to *P. vivax* infection needs to be confirmed, and this requires the Duffy antigen receptor for chemokines (DARC) (now called the atypical chemokine receptor 1, ACKR1) (33) to be present on the surface of their red cells. This requirement for DARC positivity also applies to *P. vivax* blood-stage CHMI. It is also important to ensure volunteers in mosquito bite-induced *Pv*CHMI do not have an adverse reaction to primaquine (PQ), as this is standard radical cure for dormant hypnozoites. PQ causes hemolysis in individuals deficient in glucose 6-phosphate dehydrogenase (G6PD) (47, 48); therefore, only subjects defined as having normal G6PD phenotype are recruited. In one *Pv*CHMI study (15), failure of radical cure with PQ was observed in two subjects resulting in multiple relapses. Investigations revealed that these individuals had either a non-functioning or reduced-functioning cytochrome P450 isoenzyme 2D6 (CYP2D6) genotype (“poor metabolizers” and “intermediate metabolizers” of PQ, respectively) and so were at greater risk for relapsing *P. vivax* malaria compared with those with a fully functioning CYP2D6 (“extensive metabolizers”) (49). This was a note of warning that drug failure can be difficult to predict; Oxford were subsequently able to mitigate against this by screening their volunteers for CYP2D6 genotype prior to mosquito-bite *Pv*CHMI. As a final test, they monitored participants’ sera for satisfactory clearance of PQ over 24 hours after administration of a test dose. These parameters are also of major relevance to the field as it is estimated that the combination of G6PDH deficiency and reduced functioning CYP2D6 account for nearly 40% of the population at risk of *P. vivax* infection ineligible for PQ therapy (50). For blood-stage CHMI in which hypnozoite formation does not occur, screening for G6PD and CYP2D6 can be omitted, as PQ treatment is not indicated.

## Challenge to obtain *P. vivax* blood-stage parasites to set up a blood-stage *Pv*CHMI

Infected mosquitoes are required to produce a new cryopreserved stabilate bank of *P. vivax*-infected blood. To get infected mosquitoes, a blood donation from an infected patient is required. There are two possible strategies to achieve this goal (14, 23). The first is waiting for a returning traveler from an endemic area with febrile *P. vivax* illness (**Figure 1.1**). This is the approach that the McCarthy group used to start parasite banking (14). This method is unpredictable in terms of timing and location, giving minimal notice and bringing logistical challenges for the clinical and laboratory teams, also giving no choice of patient or isolate. The second method is to produce *P. vivax*-infected mosquitoes from an endemic setting and to use the infected mosquitoes to bite volunteers (mosquito challenge-sporozoite *Pv*CHMI) to produce a blood-parasite bank for further blood-stage challenge studies. The latter allows for a more controlled and largely predictable process; however, it brings the added complication of conducting a small sporozoite *Pv*CHMI trial in carefully pre-screened “donor” individuals in order to obtain infected blood. An advantage is that different batches of mosquitoes can be selected to maximize the chance of obtaining a clonal isolate. Moreover, healthy volunteers can be carefully screened and selected in the desired (often non-endemic) country for blood group and other safety considerations so that they meet the criteria to become a safe “universal blood donor”. Lastly, the timing of production of infected mosquitoes and subsequent *Pv*CHMI can be planned in advance, so that both the clinical and laboratory teams are fully prepared. Establishment of a good insectary for malaria transmission is not that simple. Choosing the right species of vector is important and parasite-vector competency is key to ensuring a good batch of *P. vivax*-infected mosquitoes for sporozoite-induced *Pv*CHMI. The ethics committees in Thailand only allow the university to establish colonies of local vectors, but not imported species. The logistics challenge to deliver infected mosquitoes from endemic countries to the trial site in the countries, or to non-endemic countries, are much different. Ground or air transport within the country will require less complicated arrangements, requires only short-time prior notification with less documentation. The delivery of the mosquitoes to the trial sites at non-endemic countries must follow International Air Transport Association (IATA) guidelines, which have specific requirements for documents related to the infected mosquitoes, and specific packing and labeling to ensure that safety precautions are implemented. The import permit to ship the mosquitoes to institutes located in different countries will differ. In the US, the recipient is required to obtain an import permit from the US-CDC, while a letter from the recipient’s institute is required to receive the infected mosquitoes. Not all airlines will allow hand-carried infected mosquitoes into the passenger cabin and this needs to be arranged in advance. A possible alternative is to ship the infected mosquitoes *via* a commercial courier by packing them in a temperature-controlled box. This route would involve a longer transportation time from packing at the original site until arrival at the trial sites after customs clearance. In some cases, this took more than 72 h and only healthy infected mosquitoes could survive this mode of shipment. The logistical issue related to mosquito delivery will be a major concern for any trial being conducted in a non-endemic country.

## *P. vivax*-infected healthy volunteers donating blood for future blood-stage *Pv*CHMI

A further complication arises when blood from *P. vivax*-infected volunteers is used to initiate future blood-stage *Pv*CHMI studies by intravenous administration to other volunteers. Apart from passing an extensive blood-borne infection screen, eligible volunteers need to be universal blood donors (Blood Group O, Rhesus D negative, RH-). This is required to minimize the risk of any transfusion reactions occurring with future administration of their parasitized red cells (i.e., the final *P. vivax* “challenge” inoculum). Testing the blood donor’s red cells for the Kell antigen is also important if this is to be administered to female volunteers, due to the potential risk in pregnancy of developing hemolytic disease of the newborn in relation to Kell antigen incompatibility.

## Learning from recent *Pv*CHMI studies

Until early 2022, mosquito-bite induced *Pv*CHMI had been conducted in just four countries – two endemic countries, Colombia and Thailand, and two non-endemic countries, the USA and UK. Blood-stage inoculation to induce *Pv*CHMI was first established by McCarthy and team, where *P.vivax*-infected blood was banked from infected patients directly (14, 51). The group at Oxford has also established a blood stage model to induce *Pv*CHMI, but instead used a controlled parasite banking method where healthy volunteers were carefully screened and selected for mosquito-bite infection with *P. vivax* before donating infected blood for banking. The specific studies to support vaccine development are briefly described here. Information generated from these studies have help research teams to design the better trials that suit to the local research environment (local IRB, logistics and regulatory).

### Colombia

*Pv*CHMI, under modern guidelines, delivered *via* the bite of laboratory-reared, membrane-fed mosquitoes was established in the 2000s with yeoman’s work done by Herrera and colleagues in Colombia. An insectary was established to ensure access to mosquitoes prior to the first study in Cali, Colombia. After infecting mosquitoes with blood from donors in Buenaventura, they transported their mosquitoes to Cali- a distance of about 72 miles - to conduct *Pv*CHMI. In the first study (19) they performed bite-titration and established that 3 ± 1 bites resulted in a 100% infection success rate. This included the establishment of an insectary followed by establishing a reproducible infection in humans. Once established, the challenge model has been used to assess vaccine efficacy (20, 22). This is the first study to conduct the trial with less than 5 mosquito-bites. The following studies have used the standard 5 bites due to the different mosquito species—*An. albimanus* is the vector for Colombian studies, while *An. dirus* is the vector for WRAIR, Oxford and Thai studies. Arévalo-Herrera, et al, demonstrated that immunization of volunteers with *P. vivax* radiation-attenuated sporozoites (*Pv*RAS) was safe, immunogenic, and induced sterile immunity in 42% of the duffy positive volunteers in Colombia. This trial used significant numbers of volunteers for *Pv*RAS immunization compared with the trial in 1974 (52). The findings from this study confirm that immunization with *Pv*RAS is safe, immunogenic and induces sterile immunity in 42% of volunteers. This is the first study to confirm that inducing sterile protection with *Pv*RAS, as seen with *Pf*RAS, is possible. The study also identified some key immune determinants of sterile protection against *P. vivax*, which can guide the development of an effective vaccine against *P. vivax*. The detailed protocol used in this study is also published as a supplement.

### WRAIR, USA

WRAIR, in collaboration with NMRC and AFRIMS in Bangkok, began by establishing a *Pv*CHMI model in the US in 2009 to assess the efficacy of a pre-erythrocytic stage vaccine. In the US, all human clinical studies are regulated by the US-FDA, in addition to the IRB. Following extensive review and additional guidance, *Anopheles dirus* mosquitoes were fed with blood collected from infected donors in northwestern Thailand. These mosquitoes were transported to Bangkok, a distance of about 300 miles, by road. An aliquot of donor blood was also shipped to the US for blood- and vector-borne testing. Batches of mosquitoes were hand-carried to the US in secure containers following approval obtained from the CDC for the import of infectious biological agents and vectors in accordance with 42 CFR section 71.54. and approvals from the US Department of Agriculture, the US Department of Transportation, Transport Security Administration, International Air Transport Association as well as the commercial airline. Following arrival, the mosquitoes were maintained in the WRAIR insectary before the conduct of *Pv*CHMI. Only United Airlines allowed hand-carriage of the infected mosquitoes onto the plane. Recently, United Airlines stopped flying between Bangkok and the US, so that mosquito shipment by this route is no longer available. Courier shipment has been used to ship mosquitoes from Thailand to the collaborators after the trials at WRAIR in 2009. The overall duration required from packing the mosquitoes to arrival at the destination insectary was usually about 60 h, but in certain cases with delayed flight and custom clearance, this may be up to 72 h. The key success factor is the quality of the mosquitoes, as longer shipment times affect the survival rate of the mosquitoes and impact on the sporozoite development required for sporozoite-*Pv*CHM.

Two separate lots of mosquitoes infected with *P. vivax*, genotyped as Type 1(VK210) based on the CSP sequence (53), were successfully transported and used to challenge a total of 12 subjects, 6 per study (54). Following this, a third lot was used to assess the efficacy of a CSP-based vaccine (15). The last study required more coordination as the planning and immunization schedule began approximately 4 months prior to the challenge. An unexpected challenge was faced following the third study, where two subjects experienced relapses despite treatment with PQ, as previously described (15, 49). Subsequent sporozoite-induced *Pv*CHMI studies have since excluded anyone who does not have an extensive metabolizer CYP2D6 phenotype.

### Australia

The research team has established a method to prepare a *P. vivax*-infected blood bank for further intervention studies (14). This has accelerated the *Pv*CHMI, as the established protocol has shown a safe and reproducible clinical model in malaria-naive individuals. Collins et al. (51) demonstrated the safe, reproducible, and efficient transmission of *P. vivax* gametocytes from humans to mosquitoes, and established an experimental model that will accelerate the development of interventions targeting multiple stages of the *P. vivax* life cycle. More detailed protocols for steps to conduct the trial in this study were published as supplement to the paper. This provides a useful reference for other researchers who want to establish *Pv*CHMI, especially in non-endemic countries, starting with the *P. vivax* patient as blood donor. The advantage of preparing blood banking directly from *P. vivax* patients is high parasitemia. However, the large blood volume collected from symptomatic patients may raise concerns among IRBs in endemic countries for safety and feasibility to prepare blood samples for further use, as endemic populations usually stay in more remote areas with limited infrastructure and access to hospitals or public-health centers.

## Oxford, UK: How to make the parasite bank and test it for human use?

**Figure 2:**
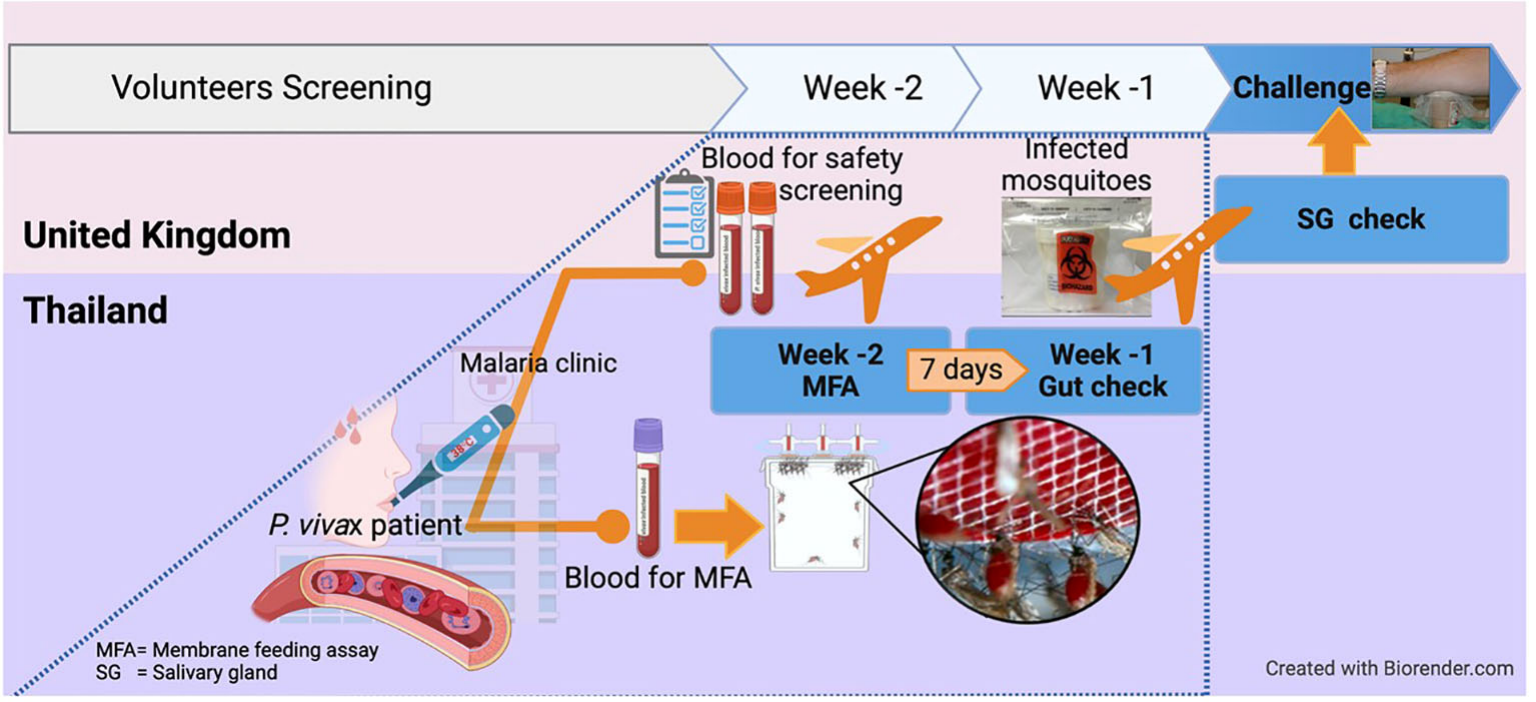
Excellent (time) match maker, the 2 weeks-notice! For mosquito-bite CHMI conducted in the UK, mosquitoes in Thailand were infected by *Plasmodium vivax-*infected patients and then shipped to the UK within 2 weeks. This is because *Plasmodium vivax* takes about 14 days for sporozoites to develop and reach the salivary glands, ready for CHMI. This also allowed time for completion of all safety laboratory tests required on the infected blood donor. Serum and whole blood samples were shipped to Oxford in real-time for extensive safety screening, including testing for blood-borne and vector-borne infections. Molecular speciation of the *P. vivax* isolate was also confirmed. The overall process required pre-organizing of all logistics to ship the patient blood and obtain the results within a week after collection, before mosquitoes were shipped on a pre-determined schedule.

To produce a cryopreserved stabilate of infected blood for *Pv*CHMI trials at Oxford, *P. vivax*-infected mosquitoes were obtained through a collaboration with the Mahidol Vivax Research Unit in Bangkok, Thailand. The whole process from identifying cases in the field and feeding mosquitoes to extensive safety, viability and clonality testing in two different countries, shipping mosquitoes to the UK and finally infecting healthy pre-screened participants by mosquito-bite *Pv*CHMI, required the alignment of multiple stars, but was completed within just 14 days, as summarized in **Figure 2**. The preparation of infected mosquitoes for Oxford and the following Mahidol studies was similar to the WRAIR study.

Following *Pv*CHMI, initiated by five infectious mosquito bites, volunteers were monitored closely for parasitemia and symptoms. On day 14 post-CHMI, both volunteers, with parasites and symptoms, were admitted to the Oxford clinical trials unit, and parasitized erythrocytes collected *via* a 250 mL blood sample (23). The challenge agent is produced stringently and safely (55, 56) following guidance on the minimum requirements for human challenge agents manufactured outside a GMP facility, and is based on principles that can be applied across high-, middle- and low-income countries (55, 57). Neither IRB in the UK or Thailand had question the manufacture of the challenge agent outside of a strict GMP setting. The UK regulators did not request to review the blood bank development protocol outside of the context of a vaccine (CTIMP) study. However, the blood banking at Oxford was manufactured in-house under “GMP-like” conditions, with full QA/QP oversight, sterile conditions and full audit trail. Thailand also does not have specific regulation for challenging agents.

Following cryopreservation of the infected blood, the process of stringent quality control testing for a number of parameters began, including sterility, mycoplasma and endotoxin. In parallel, an extensive safety screen for blood-borne infections was performed on the plasma, and an *in vitro* short-term culture viability assay was set up. Parasite DNA was then isolated and sequenced (Sanger Institute, Cambridge), to allow the analysis of leading vaccine candidate antigens and multigene families, including the vivax interspersed repeat (VIR) genes. This high-quality genome was named PvW1 and its analysis is expected to guide the future assessment of candidate vaccines and drugs, as well as experimental medicine studies (23). Only the parasite CSP gene was identified from the *P. vivax* strain used in the WRAIR trials.

Thirty-seven healthy volunteers have to-date been infected by blood-stage *Pv*CHMI with the Thai PvW1 clone with no safety concerns (Hou MM et al., unpublished data). In addition, the inoculum has been used to test the only two available clinical-stage blood-stage *P. vivax* vaccine candidates; viral-vectors ChAd63 and MVA expressing *P. vivax* Duffy-binding protein region II (PvDBPII) and protein-in-adjuvant PvDBPII in Matrix-M™ adjuvant (58, 59). As a direct result of this work, the first ever efficacy result has been obtained for a *P. vivax* blood-stage vaccine (60). The next steps include efficacy testing of this leading vaccine candidate in both naïve and exposed populations in endemic Thailand.

## Malaria infection study Thailand (MIST) Mahidol University, Thailand

The Malaria Infection Study Thailand (MIST) is underway. It commenced in 2018, and is the first in Asia. The study protocols for mosquito-bite *Pv*CHMI (MIST-1), blood injection *Pv*CHMI (MIST-2) and blood stage vaccine evaluation (MIST-3) have been adapted from the Oxford studies with some modification to meet the requirements of the local populations and Institutional Review Board concerns. The first challenge study (MIST-1) was conducted in 2019, in two volunteers, to allow production of a *P. vivax*-infected blood stabilate (Sattabongkot, unpublished data). Several rounds of consultation with local IRBs were required before submitting the protocols to both Mahidol and Oxford and obtaining IRB approval. Since Thai IRBs had rejected a similar study proposed in the late 1990s due to concerns regarding relapse in volunteers, the MIST-1 protocol included information on the relapse pattern of *P. vivax* Thai isolates and the efficacy of PQ treatment in a Thai population (61), ensuring volunteer safety and follow-up. The MIST-2 protocol was revised many times due to the uncertain impact of the Covid-19 situation on the screening of healthy volunteers before admission to the trial ward. A limitation for blood banking is that only RH+ O+ volunteers could be recruited for MIST-1 as RH- status is rare in the Thai population. With stringent inclusion/exclusion criteria required by the local IRB and the safety concerns (including allergy to insect bites, other hematological tests and CYP450 status), the likelihood of eligibility was as low as 1:6 for MIST-1. Blood-stage vaccine evaluation (MIST-3) using blood-stage challenge is dependent on completion of the MIST-2 trial, as parasite development in Thai volunteers will be used to design the MIST-3 trial.

## Discussion

Collaborative international efforts have led to the successful establishment of both sporozoite- and blood-stage *Pv*CHMI studies (14–21, 23). Despite the late start, and the complexities associated with a mosquito-bite induced *Pv*CHMI, studies in three non-endemic (UK, USA, and Australia), and two endemic countries (Colombia and Thailand) have been performed within less than 15 years, with one or both of these models (14–16, 23). The approach of producing a *P. vivax*-infected blood stabilate from healthy donors instead of patients facilitates the carefully planned and stringent production of the agent and the subsequent rigorous comparison of different vaccines or drugs by using the same *P. vivax* strain(s) across studies in different locations and populations. It also allows for the most relevant strain(s) for a particular region/population to be used. However, infection of the blood donor first requires a mosquito-bite induced *Pv*CHMI, which necessitates collaboration with teams working in endemic areas with an insectary of local vectors. Knowledge of disease epidemiology and parasite biology in different areas will help research teams plan for appropriate patient testing and optimize future *Pv*CHMI trial designs until parasite *in vitro* culture is better established. For vaccine efficacy trials that require sporozoite stage *Pv*CHMI, producing qualified *P. vivax* infected mosquitoes in endemic countries is still required until continuous culture of *P. vivax* producing infective gametocytes is established. This is the most crucial step to minimize all potential challenges to conduct *Pv*CHMI and accelerate the vaccine and drug development against *P. vivax*.

## Data availability statement

The original contributions presented in the study are included in the article/supplementary material. Further inquiries can be directed to the corresponding authors.

## Ethics statement

The studies involving human participants were reviewed and approved by VAC068 (ref 20, creation of Oxford blood bank): NCT03377296; Ethics: Approved by NRES Oxfordshire A Research Ethics Committee, ref 17/SC/0389; VAC069 (ref 20 – testing of blood bank in malaria-naïve recipients): NCT03797989; Approved by NRES South Central Hampshire A Research Ethics Committee, ref 18/SC/0577; VAC071 (ref 42 – viral-vectored DBP vaccine): NCT04009096; Approved by Oxfordshire Research Ethics Committee, ref 19/SC/0193; EudraCT (regulatory) ref 2019-000643-27; VAC079 (ref 42 – DBP protein in Matrix M vaccine): NCT04201431; Approved by Oxfordshire Research Ethics Committee A, ref 19/SC/0330; EudraCT (regulatory) ref 2019-002872-14; ForMIST1: •FTMEC: MUTM 2020-038-03, •OxTREC: 43-19, •Clinical trial registration number: NCT4083508; For MIST2: •FTMEC: MUTM 2021-047-02, •OxTREC: 15-21, •Clinical trial registration number: NCT05071079

In the review of published work from other groups, the EC approval statements can be reviewed from original publications. The patients/participants provided their written informed consent to participate in this study.

## Author contributions

All authors listed have made a substantial, direct, and intellectual contribution to the work, and approved it for publication.
